# Epidemiology, species distribution and outcome of nosocomial *Candida spp.* bloodstream infection in Shanghai

**DOI:** 10.1186/1471-2334-14-241

**Published:** 2014-05-06

**Authors:** Zhi-Tao Yang, Lin Wu, Xiao-Ying Liu, Min Zhou, Jie Li, Jia-Yin Wu, Yong Cai, En-Qiang Mao, Er-Zhen Chen, Olivier Lortholary

**Affiliations:** 1 Emergency Department & Emergency Intensive Care Unit, Ruijin Hospital, Shanghai Jiaotong University, School of Medicine, No. 197 Ruijin Er Road, Shanghai 200025, China; 2 Pôle Sino-Français de Recherches en Science du Vivant et Génomique, Ruijin Hospital, Shanghai Jiaotong University, School of Medicine, No. 197 Ruijin Er Road, Shanghai 200025, China; 3 Geriatric Department, Ruijin Hospital, Shanghai Jiaotong University, School of Medicine, No. 197 Ruijin Er Road, Shanghai 200025, China; 4 Clinical Microbiology Department, Ruijin Hospital, Shanghai Jiaotong University, School of Medicine, No. 197 Ruijin Er Road, Shanghai 200025 China; 5 Informatics Department, Ruijin Hospital, Shanghai Jiaotong University, School of Medicine, No. 197 Ruijin Er Road, Shanghai 200025, China; 6 Public Health Institute, Shanghai Jiaotong University, School of Medicine, No. 227 Chongqing Nan Road, Shanghai 200025, China; 7 Université Paris Descartes, Service des Maladies Infectieuses et Tropicales, Hôpital Necker-Enfants malades, Centre d’Infectiologie Necker Pasteur, IHU Imagine, 149, rue de Sèvres, 75743 Paris Cedex 15, France; 8 Institut Pasteur, Centre National de Référence Mycologie et Antifongiques, CNRS URA3012, Paris France

**Keywords:** *Candida spp*, Bloodstream infection, Appropriate antifungal therapy, Survival

## Abstract

**Background:**

Yeasts, mostly *Candida*, are important causes of bloodstream infections (BSI), responsible for significant mortality and morbidity among hospitalized patients. The epidemiology and species distribution vary from different regions. The goals of this study were to report the current epidemiology of *Candida* BSI in a Shanghai Teaching Hospital and estimate the impact of appropriate antifungal therapy on the outcome.

**Methods:**

From January 2008 to December 2012, all consecutive patients who developed *Candida* BSI at Ruijin University Hospital were enrolled. Underlying diseases, clinical severity, species distribution, antifungal therapy and its impact on the outcome were analyzed.

**Results:**

A total of 121 episodes of *Candida* BSI were identified, with an incidence of 0.32 episodes/1,000 admissions (0.21 in 2008 and 0.42 in 2012) The proportion of candidemia caused by non-*albicans* species (62.8%), including *C. parapsilosis* (19.8%), *C. tropicalis* (14.9%), *C. glabrata* (7.4%), *C. guilliermondii* (5.8%), *C. sake* (5.0%) was higher than that of candidemia caused by *C. albicans* (37.2%). The overall crude 28-day mortality was 28.1% and significantly reduced with appropriate empiric antifungal therapy administered within 5 days (P = 0.006). Advanced age (OR 1.04; P = 0.014), neutropenia < 500/mm^3^ (OR 17.44; P < 0.001) were independent risk factors for 28-day mortality, while appropriate empiric antifungal therapy (OR 0.369; P = 0.035) was protective against 28-day mortality.

**Conclusion:**

The epidemiology of candidemia in Shanghai differed from that observed in Western countries. Appropriate empiric antifungal therapy influenced the short-term survival.

## Background

Yeasts, mostly *Candida* spp. are currently between the fourth and the sixth most common nosocomial bloodstream isolate in American and European studies [1,2]. However, their incidences vary according to geography but overall continue to increase worldwide [3-6] except in North America and China [4,7,8]. Risk factors include exposure to broad-spectrum antibacterial agents, increased complexity of surgical procedures, prolonged use of central venous catheters, dialysis, corticosteroids and cytotoxic chemotherapy [9].

Although *C. albicans* has long been the most common species isolated during candidemia, recent studies have demonstrated a shift towards non*-albicans* species, such as *C. parapsilosis*, *C. glabrata* and *C. krusei*[3,10], especially in intensive care unit and hematological patients [11-13] and/or in case of antifungal pre-exposure [14,15]. Guidelines on the treatment of candidemia have been developed [16,17], with a consensus that all candidemic patients should receive antifungal therapy. Although novel antifungal drugs have been developed since the last decade, 90-day mortality rates due to candidemia remain as high as 50-70% [12,18,19]. In addition, data from several studies showed that 30-day mortality rates and costs of care increased significantly when empirical therapy was delayed or inadequate (inappropriate dosage, resistant isolate) [20-24].

We provide herein a laboratory-based report from a tertiary care hospital in Shanghai, China, assessing the epidemiology, species distribution, antifungal therapy and outcome of *Candida* blood stream infection (BSI). Risk factors related to 28-day crude mortality were also analyzed.

## Methods

A retrospective analysis of consecutive *Candida* bloodstream episodes in adults collected from the microbiology database of a 1300-bed tertiary care hospital in Shanghai, China was performed over a 5-year period.

Demographics, underlying diseases, co-morbidities, severity of clinical features, *Candida* species distribution, time from admission to onset and turnaround time (TAT) of final report of blood culture were compared among the patients infected with different *Candida* species.

A case of *Candida* BSI was defined as a patient with at least one blood culture positive for a *Candida*.

Definition of sepsis was signs and symptoms of inflammation plus infection with hyper- or hypothermia (core temperature >38.3°C or <36.0°C), tachycardia (90 min^−1^ or more than two SD above the normal value for age), and at least one following tachypnea or leukocytosis (WBC count > 12,000/mm^3^) or leucopenia (WBC count <4,000/mm^3^) or normal WBC count with greater than 10% immature forms.

\Neutropenia was defined as <500/mm^3^ absolute neutrophil count. Prior corticosteroid was defined as receiving >1 mg/kg/d prednisone for more than 1 week or equivalent before *Candida* BSI onset.

Isolates were detected from blood cultures using the BACTEC™ FX system (Becton Dickinson, Inc., Sparks, MD, USA). Flucytosine, amphotericin B, fluconazole, voriconazole, and itraconazole susceptibility testings were performed using the ATB® FUNGUS 3 system (BioMérieux, France), which was wildly used in China [25], providing susceptibility to antifungals results markedly concordant with those obtained using CLSI and EUCAST methodologies [26]. For triazoles, the minimal inhibitory concentration (MIC) was determined by determining the concentration at which a prominent reduction in the yeast cell count was observed after 24 h of treatment. The MIC for amphotericin B and flucytosine were defined as the lowest concentration at which no visible growth was detected. CLSI-24 h resistance breakpoints (CBPs) [27] for fluconazole, itraconazole and voriconazole, recently approved, were used: susceptible (S) ≤2 μg/mL, susceptible dose-dependent (SDD) 4 μg/mL, and resistant (R) ≥8 μg/mL for fluconazole and *C. albicans*, *C. tropicalis*, and *C. parapsilosis* and ≤32 μg/mL (SDD), ≥64 μg/mL (R) for *C. glabrata*; S ≤0.12 μg/mL, SDD 0.25-0.5 μg/mL, R ≥1 μg/mL for voriconazole and *C. albicans*, *C. tropicalis*, and *C. parapsilosis*, and ≤ 0.5 μg/mL (S), 1 μg/mL (SDD), ≥2 μg/mL (R) for *C. krusei*; S ≤ 0.12 μg/m, SDD 0.25-0.5 μg/mL, R ≥1 μg/mL for itraconazole and *C. albicans*. Due to lack of CBPs of amphotericin B and flucytosine for *Candida spp.* and itraconazole for *Candida non-albicans*, the epidemiological cutoff values (ECVs)[27] were used for them: S ≤2ug/mL, R > 2ug/mL for amphotericin B and *Candida spp.*; S ≤0.5ug/mL, R > 0.5ug/mL for flucytosine and *C. albicans*, *C. tropicalis*, *C. glabrata* and *C. parapsilosis*; S ≤32 ug/mL, R > 32ug/mL for flucytosine and *C. krusei*, S ≤1ug/mL, R > 1ug/mL for flucytosine and *C. guilliermondii*; S ≤0.5ug/mL, R > 0.5ug/mL for itraconazole and *C. parapsilosis* and *C. tropicalis*; S ≤1ug/mL, R > 1ug/mL for itraconazole and *C. krusei* and *C. guilliermondii*; S ≤2ug/mL, R > 2ug/mL for itraconazole and *C. glabrata*.

Initial appropriate antifungal treatment was considered when the appropriate drug (based on subsequent in vitro susceptibility results) with adequate dosage was started within 5 days the first blood culture performed. Adequate dosage of antifungal agent was defined according to IDSA 2009 guidelines [16]: 400-600 mg/d fluconazole for fluconazole susceptible *Candida* spp*.*; ≥0.5 mg/kg/d of amphotericin B deoxycholate; ≥3 mg/kg/d of any lipid formulation of amphotericin B; 70 mg of caspofungin × 1 dose followed by 50 mg/d and 6 mg/kg of voriconazole b.i.d. × 2 doses followed by ≥ 3 mg/kg b.i.d.. Patients who did not receive any antifungal were considered as having received inappropriate therapy.

Crude mortality was registered after 28 days from the occurrence of the episode of *Candida* BSI.

The study was approved by the local institutional review board (Ruijin Hospital, Shanghai Jiaotong University, School of medicine) and written patient consent was not required because of the observational nature of this study.

### Statistics analysis

Descriptive analyses were used for baseline characteristics and subgroup analyses (by *Candida* species). Continuous variables such as age, length of hospital stay, time from admission to infection and TAT of final results of blood culture were expressed either as means and standard deviations (SD). The Chi-square-test or 2-tailed Fisher Exact-test was used to compare categorical variables. The survival distribution function was estimated using the Kaplan-Meier method. To define risk factors for mortality, multivariate logistic regression analysis and adjusted odds ratio (OR) with 95% confidence interval (CI) were calculated. Variables that were associated with 28-day mortality in univariate analyses with a P value of < 0.16 were entered into multivariate analysis. 2-tailed tests of significance at the level of a P value of <0.05 level were used to determine statistical significance. Statistical analysis has been conducted by using the program SPSS 19.0.

## Results

### Incidence and demographic characteristics of patients

A total of 121 episodes of yeast BSI were identified during the study period (1st January 2008 to 31st December 2012), with an incidence of 0.32 episodes/1000 admissions. The incidence increased from 0.21 in 2008 to 0.42 episodes/1000 admissions in 2012 (0.21 in 2008, 0.24 in 2009, 0.26 in 2010, 0.44 in 2011 and 0.42 in 2012). The number of blood culture/1000 admissions obtained yearly in our hospital was 118.2, 116.0, 117.9, 147.6 and 166.1, respectively, while in ICUs, the number of blood culture/all blood culture yearly was 11.4%, 11.7%, 11.3%, 11.1% and 12.3%, respectively. All cases were nosocomially acquired.

**Figure 1:**
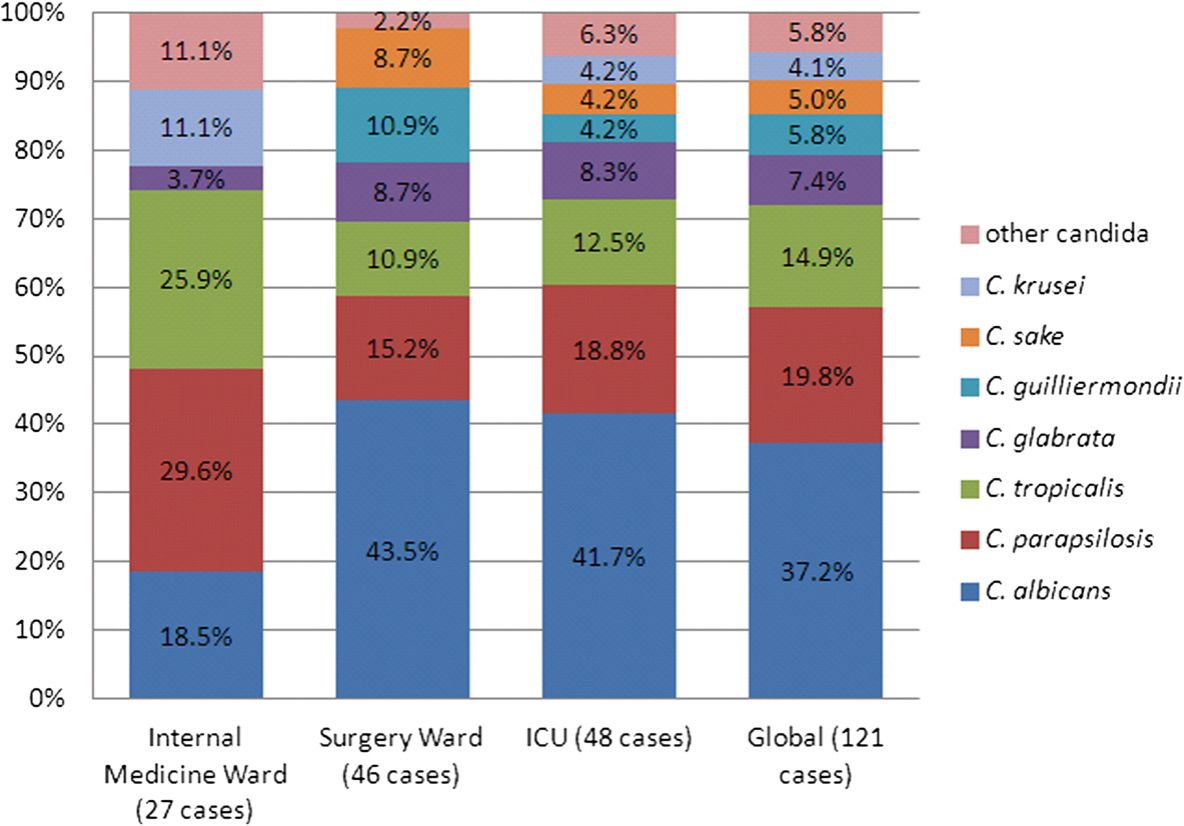
**Distribution of the *Candida *** **species by origin [*C. albicans vs. C. *** **non-*albicans *** **(p = 0.074)].**

The demographic characteristics of the patients are summarized in Table 1. The mean age of patients was 57.3 years and 71.9% were male. The distribution of *C. albicans* and non*-albicans* strains had no significant difference according to patients’ origin (p = 0.07), as shown in Figure 1. *C. albicans* was isolated in 18.5% of cases in internal medical wards while in 43.5% and 41.7% in ICU and surgery wards, respectively. However, there were a higher proportion of *C. parapsilosis* and *C. tropicalis* isolates in internal medical ward than in ICU or surgery ward. Mean TAT for *C. glabrata* (6.11 ± 1.36 d) was significantly higher than that observed for other *Candida* species (3.9-4.8 ± 0.8-1.5 d) (P = 0.002).

**Table 1:** Demographic characteristics of 121 patients with candidemia in shanghai

	***C. albicans *** **( *n *** **=45)**	***C. parapsilosis *** **( *n *** **=24)**	***C. tropicalis *** **( *n *** **=18)**	***C. glabrata (n=9)***	***C. guilliermondii (n=7)***	***C. sake (n=6)***	***C. krusei *** **( *n *** **=5)**	**Other *Candida *** (n=7)*	**Total (n=121)**	**P value**
Age (years, Mean±SD)	61.7±16.7	62.5±19.7	46.8±21.1	56.1±24.1	58.6±10.6	54.3±18.9	40.8±27.1	52.6±24.4	57.3±19.9	0.07
Male sex, *n*(%)	32(71.7)	17(70.8)	14(77.8)	9 (100)	3 (42.9)	6 (100)	2 (40.0)	4 (57.1)	87 (71.9)	-
Orginal *n*(%)										
Internal Medicine ward	5(11.2)	8(33.3)	7 (38.9)	1 (11.2)	0 (0)	0 (0)	3 (60.0)	3 (42.9)	27 (22.3)	-
Surgery ward	20(44.4)	7(29.2)	5(27.8)	4 (44.4)	5 (71.4)	4 (66.7)	0 (0)	1 (14.3)	46 (38.0)	-
Intensive care unit	20(44.4)	9(37.5)	6 (33.3)	4 (44.4)	2 (28.6)	2 (33.3)	2 (40.0)	3 (42.9)	48 (39.7)	-
Time from admission to infection	29.5±37.6	35.9±32.3	31.8±26.1	38.7±23.4	31.4±21.3	152.8±278.3	64.2±68.6	26.9±17.2	39.3±70.7	0.01
(day, Mean±SD)										
Length of hospitalization	62.1±68.6	66.0±45.4	61.1±48.8	85.0±57.4	50.7±29.7	224.3±317.8	92.4±88.5	87.9±145.7	74.6±97.7	0.02
(day, Mean±SD)										
Turnaround time (TAT)	4.04±1.36	4.83±0.82	3.94±1.43	6.11±1.36	4.43±1.13	4.33±1.75	4.40±1.52	4.86±1.22	4.44±1.38	0.002
(day, Mean±SD)										

*Other *Candida spp*. includes *C. lusitaniae* (2), *C. intermedia* (2), *C. theae* (2) and *C. haemulonii* (1).

### Underlying diseases and clinical features

The majority of patients (95.0%) had at least one co-morbidity at the time of the diagnosis of *Candida* BSI. Twenty-six patients (21.5%) had solid organ tumor, 18 (14.9%) had hematologic malignancy, 16 (13.2%) had diabetes mellitus, 12 (9.9%) had chronic renal failure, 32 (26.4%) had cardiac disease and 26 (21.5%) had chronic pulmonary disease. Twenty patients (16.5%) received corticosteroids, 66 (54.5%) underwent a surgical intervention within 30 days and 16 (13.2%) had received antifungal agents as prophylaxis or empirical therapy within 30 days prior to their onset of *Candida* BSI. Sixty-eight (56.2%) patients had at least two co-morbidities. No patient had human immunodeficiency virus (HIV) infection. Regarding the severity of clinical feature, 48 (39.7%) received at least 7-day parenteral nutrition, 22 (18.2%) were mechanically ventilated, 12 (9.9%) had renal replacement therapy, and 14 (11.6%) had absolute neutrophil count < 500/mm^3^. Patients infected with *C. parapsilosis*, *C. glabrata* or *C. krusei* had lower rate (44%-60%) of sepsis signs compared to those infected with other *Candida* spp. (more than 85%). The clinical characteristics (underlying disease, severity of clinical feature) of the patients, by *Candida* species are shown in Table 2.

**Table 2:** Clinical feature and outcome of 121 episodes of Candidemia in Shanghai

	***C. albicans *** **( *n *** **=45)**	***C. parapsilosis *** **( *n *** **=24)**	***C. tropicalis *** **( *n *** **=18)**	***C. glabrata (n=9)***	***C. guilliermondii (n=7)***	***C. sake (n=6)***	***C. krusei *** **( *n *** **=5)**	**Other *Candida *** (n=7)*	**Total (n=121)**
Underlying disease									
Solid tumor	14 (31.1)	4 (16.7)	2 (11.1)	2 (22.2)	3 (42.9)	1 (16.7)	0 (0)	0 (0)	26 (21.5)
Hematologic malignancy	3 (6.7)	2 (8.3)	6 (33.3)	1 (11.1)	0 (0)	1 (16.7)	3 (60.0)	2 (28.6)	18 (14.9)
Diabetes mellitus	5 (11.1)	5 (20.8)	1 (5.6)	3 (33.3)	0 (0)	1 (16.7)	0 (0)	1 (14.3)	16 (13.2)
Cardiac disease	13 (28.9)	5 (20.8)	3 (16.7)	4 (44.4)	2 (28.6)	2 (33.3)	2 (40.0)	1 (14.3)	32 (26.4)
Chronic pulmonary disease	13 (28.9)	4 (16.7)	2 (11.1)	2 (22.2)	1 (14.3)	1 (16.7)	1 (20.0)	2 (28.6)	26 (21.5)
Prior surgical intervention (<30 days)	28 (62.2)	10 (41.7)	9 (50.0)	5 (55.6)	6 (85.7)	4 (66.7)	2 (40.0)	2 (28.6)	66 (54.5)
Corticosteroid use	7 (15.6)	4 (16.7)	2 (11.1)	2 (22.2)	0 (0)	0 (0)	3 (60.0)	2 (28.6)	20 (16.5)
Prior antifungal agents use (<30 days)	1 (2.2)	5 (20.8)	2 (11.1)	2 (22.2)	1 (14.3)	1 (16.7)	4 (80.0)	0 (0)	16 (13.2)
Severity of clinical feature									
Parenteral nutrition	20 (44.4)	9 (37.5)	3 (16.7)	7 (77.8)	3 (42.9)	2 (33.3)	2 (40.0)	2 (28.6)	48 (39.7)
Mechanical ventilation	9 (20.0)	4 (16.7)	1 (5.6)	5 (55.6)	0 (0)	1 (16.7)	2 (40.0)	0 (0)	22 (18.2)
Sepsis	40 (88.9)	14 (58.3)	17 (94.4)	4 (44.4)	6 (85.7)	5 (83.3)	3 (60.0)	6 (85.7)	95 (78.5)
Renal replacement therapy	6 (13.3)	2 (8.3)	0 (0)	2 (22.2)	1 (14.3)	0 (0)	1 (20.0)	0 (0)	12 (9.9)
Central venous catheter	37 (82.2)	16 (66.7)	13 (72.2)	9 (100)	6 (85.7)	4 (66.7)	4 (80.0)	6 (85.7)	95 (78.5)
Neutropenia	2 (4.4)	0 (0)	6 (33.3)	1 (11.1)	0 (0)	0 (0)	3 (60.0)	2 (28.6)	14 (11.6)
28-day mortality	16 (35.6)	4 (16.7)	7 (38.9)	0 (0)	1 (14.3)	0 (0)	2 (40.0)	4 (57.1)	34 (28.1)

*Other *Candida spp*. includes *C. lusitaniae* (2), *C. intermedia* (2), *C. theae* (2) and *C. haemulonii* (1).

### *Candida* species and antifungal susceptibility testing

Forty-five (37.2%) of *Candida* BSI were *C. albicans* and 76 (62.8%) were non-*albicans Candida.* In non-*albicans Candida* sepsis cases, 24 (19.8%) were caused by *C. parapsilosis*, 18 (14.9%) by *C. tropicalis*, 9 (7.4%) by *C. glabrata*, 7 (5.8%) by *C. guilliermondii*, 6 (5.0%) by *C. sake*, 5 (4.1%) by *C. krusei*, 2 (1.7%) by *C. lusitaniae*, 2 (1.7%) by *C. intermedia*, 2 (1.7%) by *C. theae* and 1 (0.8%) by *C. haemulonii*.

Based on CLSI breakpoints 2012 (CBPs) [27], the rate of susceptibility to fluconazole was 93.0%, 95.8%, 66.7%, 85.7%, 100% and 0% for *C. albicans*, *C. parapsilosis*, *C. tropicalis*, *C. guilliermondii*, *C. sake* and *C. krusei*, respectively. The rate of susceptibility to itraconazole was lower in *C. albicans* in our study (79.1%) when compared to fluconazole (93.0%) and voriconazole (95.3%). Amphotericin B and 5-flucytosine remained close to 100% susceptibility against common *Candida spp.*, except for *C. krusei* and *C. guilliermondii* (only 20% and 71.4% susceptible to flucytosine, respectively), according to ECVs [27] (showed in Table 3).

**Table 3:** **Antifungal susceptibility testing results (ATB Fungus 3) of 105 Candida isolates in Shanghai according to new species-specific clinical breakpoints (CBPs) or epidemiologic cutoff values (ECVs) defined by the CLSI**[27]

	***C. albicans *** **(n=43)**	***C. parapsilosis *** **(n=24)**	***C. tropicalis *** **(n=18)**	***C. glabrata *** **(n=8)**	***C. guilliermondii *** **(n=7)**	***C. krusei *** **(n=5)**
Fluconazole						
Susceptible	40 (93.0)	23 (95.8)	12 (66.7)	0 (0)	6 (85.7)	0 (0)
SDD	0 (0)	0 (0)	1 (5.5)	8 (100)	0 (0)	0 (0)
Resistance	3 (7.0)	1 (4.2)	5 (27.8)	0 (0)	1 (14.3)	5 (100)
Itraconazole						
Susceptible	34 (79.1)	23 (95.8)	11 (61.1)	4 (50.0)	5 (71.4)	1 (20.0)
SDD	5 (11.6)	-	-	-	-	-
Resistance	4 (9.3)	1 (4.2)	7 (38.9)	4 (50.0)	2 (28.6)	4 (80.0)
Voriconazole						
Susceptible	41 (95.3)	23 (95.8)	13 (72.2)	8 (100)	5 (71.4)	4 (80.0)
SDD	0 (0)	0 (0)	1 (5.6)	0 (0)	0 (0)	0 (0)
Resistance	2 (4.7)	1 (4.2)	4 (22.2)	0 (0)	2 (28.6)	1 (20.0)
Amphotericin B						
Susceptible	43 (100)	24 (100)	18 (100)	8 (100)	6 (85.7)	5 (100)
Resistance	0 (0)	0 (0)	0 (0)	0 (0)	1 (14.3)	0 (0)
Flucytosine						
Susceptible	42 (97.7)	24 (100)	18 (100)	8 (100)	5 (71.4)	1 (20.0)
Resistance	1 (2.3)	0 (0)	0 (0)	0 (0)	2 (28.6)	4 (80.0)

SDD: susceptible dose dependence.

Due to lack of CBPs of amphotericin B and flucytosine for *Candida spp.* and itraconazole for *Candida non-albicans*, the epidemiological cutoff values (ECVs) were used [27].

### Antifungal therapy and outcome

Empiric antifungal therapy was administered in 97 (80.2%) of the cases while 24 (19.8%) patients did not receive any antifungal treatment. Fluconazole was most frequently used (62.9%), followed by caspofungin (18.5%) and any formulation of amphotericin B for (6.2%). Four patients (4.1%) received a combination therapy (fluconazole + caspofungin and fluconazole + amphotericin B in 2 cases each). Eighty-six (71.1%) of 121 patients with *Candida* BSI received an appropriate empiric antifungal agent.

**Figure 2:**
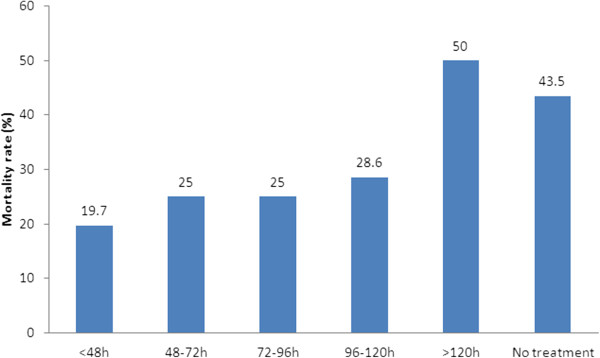
**Relationship between hospital mortality (28-day) and the timing of antifungal treatment.** The timing of antifungal therapy was determined to be from the time when the first blood sample for culture that was positive for fungi was drawn to the time when appropriate antifungal treatment was first administered to the patient. 28-day mortality was significantly lower for those who received appropriate empiric antifungal therapy within 120 h (5d) *vs*. inappropriate or delayed (>5d) empiric antifungal therapy or no antifungal treatment (20.9% *vs*. 45.7%, p = 0.006).

**Figure 3:**
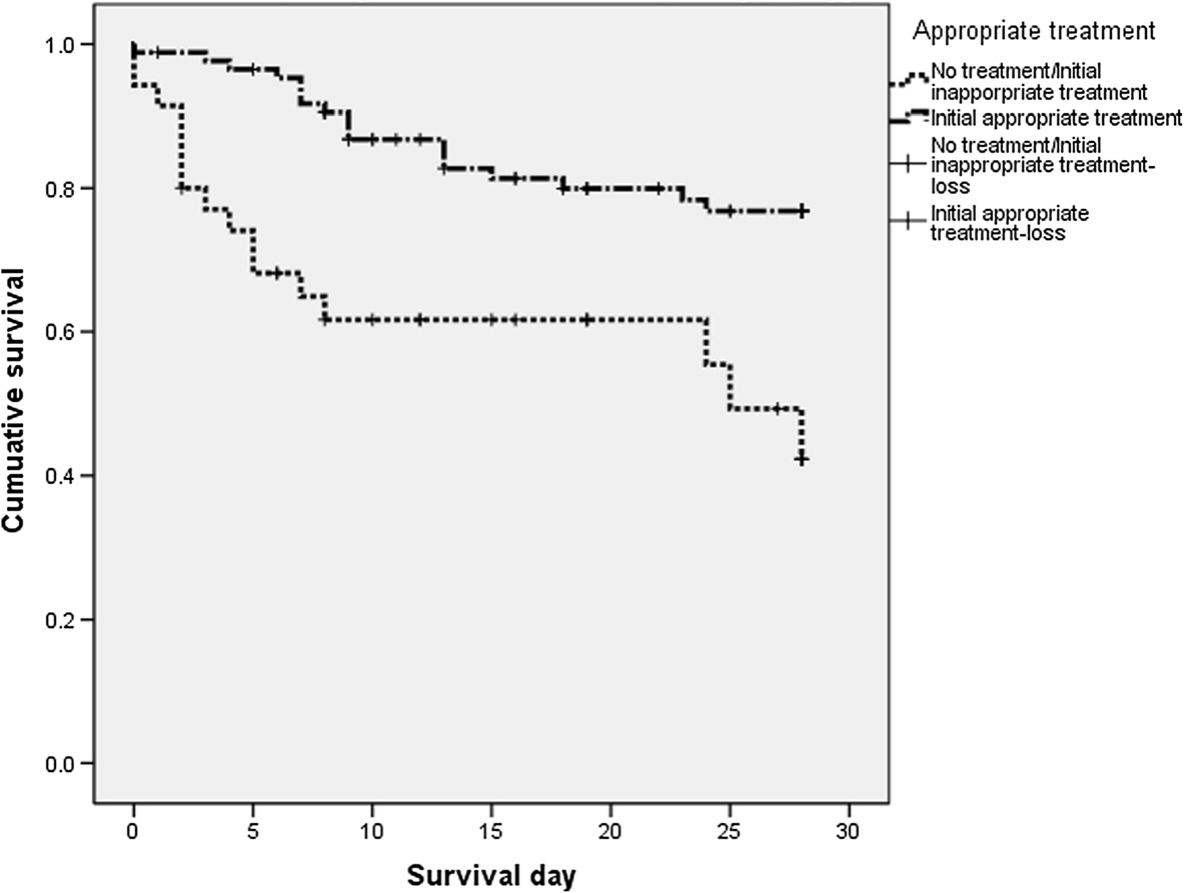
**Kaplan–Meier survival curves (28-day) based on the initiation of empiric appropriate antifungal therapy [Log Rank (mantel-Cox) chi-square =12.784, *p*** **< 0.001].**

Patient outcomes at 28-day, stratified by yeast species, are reported in Table 2. The overall, 28-day crude mortality rate was 28.1%. Mortality rate was significantly higher in internal medicine than in surgical wards or in ICU (48.1% vs. 17.4% or 27.1%, respectively, p = 0.018). Though still considerable, mortality was significantly lower for those who received appropriate empiric antifungal therapy than inappropriate empiric antifungal therapy (20.9% *vs.* 45.7%, p = 0.006) within 5 days the first positive blood culture performed (shown in Figure 2). There was a significant difference in survival between the two groups [Log Rank (mantel-Cox) chi-square =12.784, P < 0.001] (shown in Figure 3).

In univariate analysis, gender (male), presence of sepsis, neutropenia and appropriate empiric antifungal therapy were associated with 28-day mortality. In multivariate analysis, advanced age (OR, 1.038; 95% CI, 1.007-1.069; P = 0.014), neutropenia (OR, 17.442; 95% CI 3.555-85.582; P < 0.001) and appropriate empiric antifungal therapy (OR, 0.369; 95% CI 0.146-0.934; P = 0.035) remained independent risk factors for 28-day mortality (Table 4).

**Table 4:** Risk factors for 28-day mortality of candidemia in Shanghai (n=121 episodes)

	**28-day outcome**			**Mutlivariate analysis**	
	**Survival (n=87)**	**Death (n=34)**	**P value**	**OR (95% CI)**	**P value**
Gender, male [*n*(%)]	67 (77.0)	20 (58.8)	0.045	-	0.25
Age (years, Mean±SD)	55.70±20.71	61.38±17.29	0.159	1.038 (1.007-1.069)	0.014
Underlying disease					
Solid tumor [*n*(%)]	15 (17.2)	11 (32.4)	0.069	-	0.177
Hematologic malignancy [*n*(%)]	10 (11.5)	8 (23.5)	0.094	-	0.313
Diabetes mellitus [*n*(%)]	10 (11.5)	6 (17.6)	0.369	-	-
Cardiac disease [*n*(%)]	26 (29.9)	6 (17.60	0.17	-	-
Pulmonary disease [*n*(%)]	17 (19.5)	9 (26.5)	0.404	-	-
Prior surgical intervention (<30 days) [*n*(%)]	51 (58.6)	15 (44.1)	0.15	-	0.806
Prior antifungal agents use (<30 days) [*n*(%)]	13 (14.9)	3 (8.8)	0.372	-	-
Corticosteroid use [*n*(%)]	13 (14.9)	7 (20.6)	0.452	-	-
Severity of clinical feature					
Sepsis [*n*(%)]	64 (73.6)	31 (91.2)	0.034	-	0.087
Renal replacement therapy [*n*(%)]	7 (8.0)	5 (14.7)	0.445	-	-
Parenteral nutrition [*n*(%)]	34 (39.1)	14 (41.2)	0.832	-	-
Mechanical ventilation [*n*(%)]	15 (17.2)	7 (20.6)	0.668	-	-
Central venous catheter [*n*(%)]	68 (78.2)	27 (79.4)	0.88	-	-
Neutropenia [*n*(%)]	5 (5.7)	9 (26.5)	0.004	17.442 (3.555-85.582)	<0.001
Antifungal therapy within 5 days the first blood culture performed					
Appropriate empiric therapy	68 (78.2)	18 (52.9)	0.006	0.369 (0.146-0.934)	0.035
Inappropriate empiric therapy	19 (21.8)	16 (47.1)			

## Discussion

Our data show that in our teaching hospital in Shanghai, the incidence of candidemia has increased steadily in the past 5 years (0.21 to 0.42 episodes per 1,000 admissions). Several studies have shown a substantial increase in the incidence of candidemia in the past 2 decades [3-5], with an incidence of 0.3-1.0 episodes per 1000 admissions elsewhere in China [28], North America [29,30] and some European countries [3,31,32], much higher (1.2 to 1.7 episodes) in Latin America [33] and Italy [3], and much lower in Northern Europe (0.01 to 0.08 episodes) [34,35].

Species distribution here consisted in *C. albicans*, followed by *C. parapsilosis*, *C. tropicalis* and *C. glabrata,* overall accounting for more than 70% of episodes of candidemia. Although *C. albicans* is still considered the most common species causing candidemia, trends of increasing rates of non*-C. albicans* have been reported worldwide [3,10,36], which was also observed in our study (37.2% for *C. albicans vs.* 62.8% for non-*C. albicans*). The reasons for the emergence of non-*C. albicans* species are not fully understood, but some studies recently showed that medical conditions may influence the risk of developing non-*C. albicans* invasive infection. Indeed, *C. parapsilosis* candidemia has been associated with vascular catheters, parenteral nutrition and prior exposure to echinocandins [11]. *C. tropicalis* is associated with older age, cancer and neutropenia [37,38], and *C. krusei* and *C. glabrata* are associated with previous exposure to azoles and echinocandins [39,40]. Although no statistical analysis was performed here on the interaction between patient’s clinical features and *Candida* species, because of small sample size, the trends of distribution are partially supportive of these reports: 33-60% patients infected with *C. tropicalis* or *C. krusei* had hematologic malignancy and neutropenia; four of five (80%) patients infected with *C. krusei* had prior use of antifungal therapy; 40-55% patients infected with *C. glabrata* or *C. krusei* received mechanical ventilation. Of note, our study showed that presence of sepsis symptoms was predominant in patients infected with *C. albicans* (88.9%), *C. tropicalis* (94.4%), *C. guilliermondii* (85.7%) and *C. sake* (83.3%) rather than in patients infected by other *Candida* species (44.4%-60.0%). Interestingly, we observed in the present study that *C. guilliermondii* and *C. sake* represented 5.8% and 5.0% of the isolates; while only 0.2%-2.4% of *C. guilliermondii* was observed in European countries [11,18,41,42], in Taiwan [43] and in Latin America except in Honduras (20%) [33]. The emergence of severe sepsis due to *C. sake* is here remarkable, knowing that the latter species represented only 0.03% of isolates in a recent publication from ARTEMIS [44].

A lower percentage of *C. albicans* (18.5%) was observed in internal medicine wards compared with surgical wards (43.5%) and ICU (41.7%) while *C. tropicalis* and *C. parapsilosis* were more frequent in internal medicine wards.

Antifungal resistance was a rare finding in our study and was restricted to azoles, except in *C. tropicalis*. We found a higher rate of fluconazole SDD or resistance *C. tropicalis* here (5.5% and 27.8%, respectively), while other studies showed a prevalence of less than 10% [4,33,45-47].

In the present study, almost 19.8% of patients did not receive any antifungal treatment. The most likely reasons were late diagnosis, since one-third of these patients died within 7 days after the onset candidemia, and the poor compliance to antifungal therapy in some wards. Fluconazole was the most frequently administered antifungal agents as empiric therapy, followed by the echinocandins. Echinocandins were used infrequently despite their efficacy in the treatment of candidemia and their recommendation to be used as first line therapy in the recent ESCMID guidelines [17,48], because of their price compared with fluconazole.

We report an overall, 28-day crude mortality rate of 28.1%. A statistically significant finding is the high 28-day mortality rate for *Candida* BSI in patients from internal medicine wards compared with other wards. The factors contributing towards poor short-term survival in internal medicine should be explored in future studies.

Another major finding in the present study was that delay in initial appropriate empirical treatment for *Candida* BSI of at least 5 days after the first blood culture was performed was associated with a greater risk of 28-day mortality. Our results confirm recent studies’ results, in which a relationship between delaying the initiation of antifungal therapy and mortality was demonstrated [24,49]. For example, Bassetti *et al.*[49] showed that the initiation of antifungal treatment 48 h after having the first positive blood culture report was an independent determinant of hospital mortality. The results of previous studies on this topic have yielded conflicting results [20,21,50]. Hsu *et al.*[50] investigated patients who received an echinocandin for *Candida* BSI; early (<3 days) versus late (>3 days) initiation of caspofungin was not associated with hospital mortality. Parkins *et al.*[20] showed although appropriate empirical antifungal therapy was protective against hospital mortality, no association was found between timing of antifungal administration and mortality. Similarly, Kludze-Forson *et al.*[21] reported the lack of any association between the timing of appropriate antifungal therapy and hospital mortality; of note, increased APACHE II score was the only independent predictor of mortality.

Reasons for observing different results among various studies investigating the impact of timing of antifungal therapy on outcomes is unknown, but multiple complex factors are likely responsible. Our study showed in multivariate logistic regression analysis, the significant association between 3 variables (age, neutropenia and appropriate empiric antifungal therapy) and 28-day mortality. Appropriate empiric antifungal therapy was here a protective factor.

Although providing data from a large university hospital in Shanghai, our study has some important limitations, the most significant being its retrospective nature. The severity of illness score (such as APACHE II score), timing of CVC removal were not included because of missing data. Second, our study was monocentric and the results may not be applicable to other settings.

## Conclusions

In conclusion, our retrospective study showed the incidence of candidemia has increased in the past 5 years in Shanghai. We observed a predominance of non-*C. albicans Candida* species, with *C. guilliermondii* and *C. sake* representing more than 10% of the isolates. However *C. albicans* remained the most frequently isolated species. 28-day mortality in internal medicine wards was unacceptably high. Finally, appropriate empiric antifungal therapy influenced the short-term survival of patients with candidemia.

### Key messages

The incidence of candidemia has increased in the past 5 years at Ruijin hospital in Shanghai.

*C. albicans* remained the most frequently isolated species, while a predominance of non-*C. albicans Candida* species was observed, with *C. guilliermondii* and *C. sake* representing more than 10% of the isolates.

Twenty-eight-day mortality rate was significantly higher in internal medicine than in surgical wards or in ICU.

Advanced age, neutropenia and appropriate empiric antifungal therapy remained independent risk factors for 28-day mortality.

## Competing interests

The authors declare that they have no competing interests.

## Authors’ contributions

ZTY, LW, EZC and OL made substantial contributions to conception and design. ZTY, LW and XYL participated in acquisition of data. ZTY and YC participated in data analysis. ZTY and LW drafted the manuscript. EZC and OL revised the multiple versions of the manuscript critically. All authors read and approved the final manuscript.

## Pre-publication history

The pre-publication history for this paper can be accessed here:

http://www.biomedcentral.com/1471-2334/14/241/prepub
